# (Benzyl­amine)chloridobis(ethane-1,2-diamine)cobalt(III) dichloride hemihydrate

**DOI:** 10.1107/S1600536809034849

**Published:** 2009-09-05

**Authors:** K. Ravichandran, P. Ramesh, M. Tamilselvan, K. Anbalagan, M. N. Ponnuswamy

**Affiliations:** a Centre of Advanced Study in Crystallography and Biophysics, University of Madras, Guindy Campus, Chennai 600 025, India; b Department of Chemistry, Pondicherry University, Puducherry 605 014, India

## Abstract

In the title compound, [CoCl(C_2_H_8_N_2_)_2_(C_7_H_9_N)]Cl_2_·0.5H_2_O, there are two crystallographically independent cations and anions and one water mol­ecule in the asymmetric unit. Both Co^III^ ions are bonded to two chelating ethylenediamine ligands, one benzylamine molecule and one chloride ion. The crystal packing is through N—H⋯O, N—H⋯Cl and O—H⋯Cl inter­actions.

## Related literature

For the importance of metal complexes in the fields of bio­logical catalysis and functions, see: Gray (2003 ▶); Wohrle & Pomogailo (2003 ▶). For the biomedical applications of cobalt complexes, see: Osinsky (2004 ▶); Roth *et al.* (2002 ▶). For puckering and asymmetry parameters, see: Cremer & Pople (1975 ▶); Nardelli (1983 ▶). For related structures, see: Lee *et al.* (2007 ▶); Ramesh *et al.* (2008 ▶). *cis*-[Co^III^(en)_2_(BzNH_2_)Cl]Cl_2_·0.5H_2_O was synthesized (Bailer & Clapp, 1945 ▶) by substituting the chloride ligand with benzyl amine in *trans*-[Co(en)_2_Cl_2_]Cl (Bailer & Rollinson, 1946 ▶).
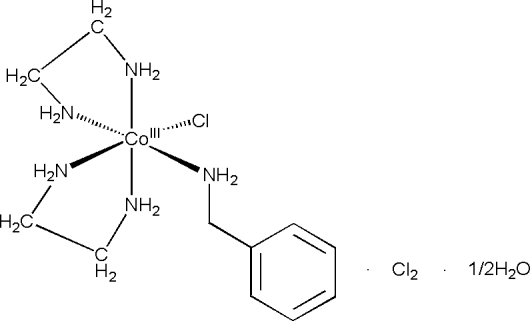


## Experimental

### 

#### Crystal data

[CoCl(C_2_H_8_N_2_)_2_(C_7_H_9_N)]Cl_2_·0.5H_2_O
*M* *_r_* = 401.65
Monoclinic, 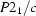

*a* = 20.9361 (9) Å
*b* = 7.2447 (3) Å
*c* = 24.4340 (9) Å
β = 106.440 (2)°
*V* = 3554.5 (3) Å^3^
*Z* = 8
Mo *K*α radiation
μ = 1.42 mm^−1^
*T* = 293 K
0.25 × 0.20 × 0.20 mm

#### Data collection

Bruker Kappa APEXII area-detector diffractometer
Absorption correction: multi-scan (*SADABS*; Sheldrick, 2001 ▶) *T*_min_ = 0.718, *T*_max_ = 0.765
40140 measured reflections
8911 independent reflections
6475 reflections with *I* > 2σ(*I*)
*R*_int_ = 0.044

#### Refinement

*R*[*F* ^2^ > 2σ(*F* ^2^)] = 0.037
*wR*(*F* ^2^) = 0.096
*S* = 1.06
8911 reflections
458 parameters
2 restraints
H atoms treated by a mixture of independent and constrained refinement
Δρ_max_ = 0.61 e Å^−3^
Δρ_min_ = −0.41 e Å^−3^

### 

Data collection: *APEX2* (Bruker, 2004 ▶); cell refinement: *SAINT* (Bruker, 2004 ▶); data reduction: *SAINT*; program(s) used to solve structure: *SHELXS97* (Sheldrick, 2008 ▶); program(s) used to refine structure: *SHELXL97* (Sheldrick, 2008 ▶); molecular graphics: *ORTEP-3* (Farrugia, 1997 ▶); software used to prepare material for publication: *SHELXL97* and *PLATON* (Spek, 2009 ▶).

## Supplementary Material

Crystal structure: contains datablocks global, I. DOI: 10.1107/S1600536809034849/bt5046sup1.cif

Structure factors: contains datablocks I. DOI: 10.1107/S1600536809034849/bt5046Isup2.hkl

Additional supplementary materials:  crystallographic information; 3D view; checkCIF report

## Figures and Tables

**Table 1:** Hydrogen-bond geometry (Å, °)

*D*—H⋯*A*	*D*—H	H⋯*A*	*D*⋯*A*	*D*—H⋯*A*
N1—H1*A*⋯Cl3	0.81 (3)	2.67 (3)	3.406 (2)	151 (3)
N1—H1*B*⋯Cl2	0.87 (3)	2.36 (3)	3.211 (2)	163 (2)
N8—H8*A*⋯Cl3	0.91 (3)	2.33 (3)	3.179 (2)	156 (3)
N4′—H4*D*⋯O1	0.81 (3)	2.25 (3)	2.915 (3)	139 (2)
N8′—H8*C*⋯O1	0.87 (3)	2.15 (3)	2.964 (4)	155 (3)
N1′—H1*C*⋯Cl3′	0.82 (3)	2.43 (4)	3.246 (2)	174 (3)
N4—H4*A*⋯Cl2^i^	0.87 (3)	2.65 (3)	3.423 (2)	149 (2)
N5′—H5*D*⋯Cl2′	0.85 (3)	2.39 (3)	3.233 (2)	168 (3)
N8′—H8*D*⋯Cl3′	0.87 (3)	2.48 (3)	3.252 (2)	148 (3)
N4—H4*B*⋯Cl3^ii^	0.93 (3)	2.45 (3)	3.265 (2)	147 (2)
N8—H8*B*⋯Cl3^ii^	0.79 (3)	2.49 (3)	3.280 (2)	176 (3)
N9—H9*A*⋯Cl2^iii^	0.84 (4)	2.57 (4)	3.391 (2)	166 (3)
N5′—H5*C*⋯Cl1^i^	0.91 (3)	2.63 (3)	3.380 (2)	141 (2)
N1′—H1*D*⋯Cl2′^iv^	0.89 (3)	2.49 (3)	3.288 (2)	149 (2)
N9′—H9*D*⋯Cl2′^iv^	0.85 (3)	2.81 (3)	3.615 (2)	158 (2)
N5—H5*A*⋯Cl2^i^	0.88 (3)	2.38 (3)	3.220 (2)	159 (2)
O1—H2*W*⋯Cl3′^i^	0.842 (17)	2.27 (2)	3.092 (3)	165 (4)

Symmetry codes: (i) 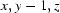; (ii) 
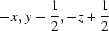
; (iii) 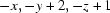; (iv) 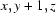.

**Fig. 1.**
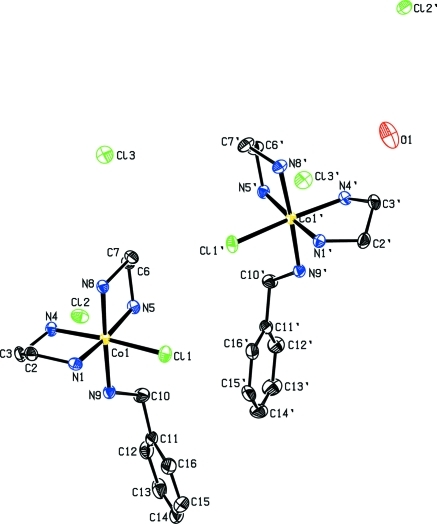
Perspective view of the asymmetric unit of the title compound. Displacement ellipsoids are drawn at the 30% probability level. The H atoms are omitted for clarity.

**Fig. 2.**
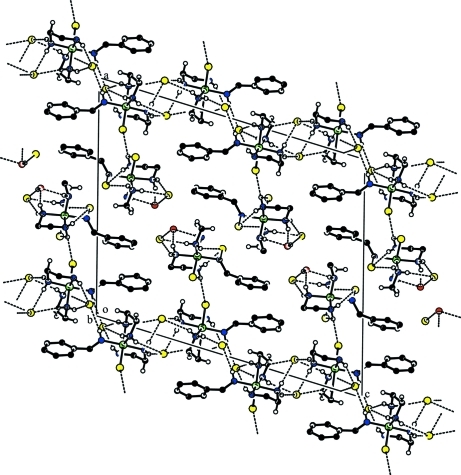
The crystal packing of the molecules viewed down the b–axis. H atoms not involved in hydrogen bonding have been omitted for clarity.

## supplementary crystallographic information

### Comment

Metal complexes find importance in the fields of biological catalysis and functions, such as in metabolism (Gray, 2003; Wohrle & Pomogailo, 2003). Cobalt complexes were also found to show biomedical applications and one such example is cancer therapy (Osinsky, 2004; Roth *et al.*, 2002). Against this background and to ascertain the molecular conformation, the structure determination of the title compound has been carried out.

There are two crystallographically independent molecules in the asymmetric unit. The Co^III^ ion and the four N atoms almost lie in the same plane, whereas the other N and Cl atoms are approximately perpendicular to this plane. The Co—N and Co—Cl bond lengths are comparable with the related complexes (Lee *et al.*,  2007; Ramesh *et al.*,  2008). In the molecule A, the two five membered rings adopt twist conformation with the puckering parameters (Cremer & Pople, 1975) and the asymmetry parameters (Nardelli, 1983) [for the ring Co1/N1/C2/C3/N4 are: q_2_ = 0.416 (3) Å, φ= 90.4 (3)° and Δ2(Co1)= 0.10 (2)°; and for the ring Co1/N5/C6/C7/N8 are: q_2_ = 0.445 (3) Å, φ= 90.7 (3)° and Δ2(Co1)= 1.1 (2)°]. One of the five membered rings in the molecule B adopts twist conformation, whereas the other ring adopts envelope conformation [for the ring Co1'/N1'/C2'/C3'/N4' are: q_2_ =0.393 (2) Å, φ= 89.0 (3)° and Δ2(Co1')= 1.2 (2)°; and for the ring Co1'/N5'/C6'/C7'/N8' are: q_2_ = 0.443 (3) Å, φ= 281.0 (3)° and Δ2(Co1')= 11.1 (1)°].

The crystal packing is controlled by N—H···O, N—H···Cl, O—H···Cl and C—H···π types of intra and intermolecular interactions. The two intra molecular N—H···O hydrogen bonds form a S(6) ring motif. The combination of N4'-H4'D···O1, N8'-H8D···Cl3' and O1—H2W···Cl3' hydrogen bonds connects the molecule into one dimensional chain running along b–axis.

### Experimental

Cis-[Co^III^(en)_2_(BzNH_2_)Cl]Cl_2_.1/2 H_2_O was synthesized (Bailer and Clapp, 1945), by substituting chloride ligand with benzyl amine (Bz) in *trans*- [Co(en)_2_Cl_2_]Cl (Bailer and Rollinson, 1946). Two grams of the cobalt^(III)^ complex was suspended in 1 ml of water in a mortar. To this a definite amount of AnalaR benzylamine was added in drops with constant grinding to obtain a paste. A rosy red color was observed and the grinding was continued for another 1 hr to obtain a homogeneous solid mass. The paste was then allowed to stand overnight in a desicator. The Bz substituted complex was recrystallized twice using acidified water, dried and preserved in a desicator. Single crystal was grown by adding the metal complex in triply distilled water acidified with HCl and kept standing at 0°C for 2–3 weeks.

### Refinement

Nitrogen and Oxygen H atoms were refined and other H atoms were positioned geometrically (C—H=0.93–0.97 Å) and allowed to ride on their parent atoms, with 1.2*U*_eq_(C).

### Crystal data

**Table:**

[CoCl(C_2_H_8_N_2_)_2_(C_7_H_9_N)]Cl_2_·0.5H_2_O	*F*(000) = 1672
*M**_r_* = 401.65	*D*_x_ = 1.501 Mg m^−^^3^
Monoclinic, *P*2_1_/*c*	Mo *K*α radiation, λ = 0.71073 Å
Hall symbol: -P 2ybc	Cell parameters from 4562 reflections
*a* = 20.9361 (9) Å	θ = 1.0–28.4°
*b* = 7.2447 (3) Å	µ = 1.42 mm^−^^1^
*c* = 24.4340 (9) Å	*T* = 293 K
β = 106.440 (2)°	Block, pink
*V* = 3554.5 (3) Å^3^	0.25 × 0.20 × 0.20 mm
*Z* = 8	

### Data collection

**Table:**

Bruker Kappa APEXII area-detector diffractometer	8911 independent reflections
Radiation source: fine-focus sealed tube	6475 reflections with *I* > 2σ(*I*)
graphite	*R*_int_ = 0.044
ω and φ scans	θ_max_ = 28.4°, θ_min_ = 1.0°
Absorption correction: multi-scan (*SADABS*; Sheldrick, 2001)	*h* = −28→27
*T*_min_ = 0.718, *T*_max_ = 0.765	*k* = −9→9
40140 measured reflections	*l* = −32→32

### Refinement

**Table:**

Refinement on *F*^2^	Primary atom site location: structure-invariant direct methods
Least-squares matrix: full	Secondary atom site location: difference Fourier map
*R*[*F*^2^ > 2σ(*F*^2^)] = 0.037	Hydrogen site location: inferred from neighbouring sites
*wR*(*F*^2^) = 0.096	H atoms treated by a mixture of independent and constrained refinement
*S* = 1.06	*w* = 1/[σ^2^(*F*_o_^2^) + (0.0439*P*)^2^ + 1.0235*P*] where *P* = (*F*_o_^2^ + 2*F*_c_^2^)/3
8911 reflections	(Δ/σ)_max_ = 0.003
458 parameters	Δρ_max_ = 0.61 e Å^−^^3^
2 restraints	Δρ_min_ = −0.41 e Å^−^^3^

### Special details

**Table:**

Geometry. All e.s.d.'s (except the e.s.d. in the dihedral angle between two l.s. planes) are estimated using the full covariance matrix. The cell e.s.d.'s are taken into account individually in the estimation of e.s.d.'s in distances, angles and torsion angles; correlations between e.s.d.'s in cell parameters are only used when they are defined by crystal symmetry. An approximate (isotropic) treatment of cell e.s.d.'s is used for estimating e.s.d.'s involving l.s. planes.
Refinement. Refinement of *F*^2^ against ALL reflections. The weighted *R*-factor *wR* and goodness of fit *S* are based on *F*^2^, conventional *R*-factors *R* are based on *F*, with *F* set to zero for negative *F*^2^. The threshold expression of *F*^2^ > σ(*F*^2^) is used only for calculating *R*-factors(gt) *etc*. and is not relevant to the choice of reflections for refinement. *R*-factors based on *F*^2^ are statistically about twice as large as those based on *F*, and *R*- factors based on ALL data will be even larger.

### Fractional atomic coordinates and isotropic or equivalent isotropic displacement parameters (Å^2^)

**Table:**

	*x*	*y*	*z*	*U*_iso_*/*U*_eq_	
Co1	0.086901 (14)	0.76169 (4)	0.397792 (12)	0.02243 (8)	
Co1'	0.378670 (14)	0.24118 (4)	0.376673 (12)	0.02283 (8)	
Cl1	0.18662 (3)	0.89958 (10)	0.40924 (3)	0.04223 (16)	
Cl1'	0.28589 (3)	0.41206 (9)	0.36007 (3)	0.04156 (16)	
Cl2	0.03854 (4)	1.27863 (9)	0.47345 (3)	0.04187 (16)	
Cl2'	0.44225 (3)	−0.22621 (9)	0.47024 (3)	0.03979 (16)	
Cl3	0.07194 (4)	1.07730 (10)	0.24584 (3)	0.04584 (17)	
Cl3'	0.40208 (4)	0.54666 (10)	0.23461 (3)	0.04853 (18)	
O1	0.46177 (15)	−0.0796 (4)	0.28551 (13)	0.0771 (8)	
H1W	0.4962 (16)	−0.050 (6)	0.2760 (17)	0.091 (15)*	
H2W	0.453 (2)	−0.189 (3)	0.2743 (17)	0.090 (14)*	
N1	0.04245 (11)	0.9940 (3)	0.37372 (10)	0.0301 (5)	
H1A	0.0583 (14)	1.045 (4)	0.3509 (12)	0.040 (8)*	
H1B	0.0506 (12)	1.073 (4)	0.4018 (11)	0.030 (7)*	
N1'	0.42817 (10)	0.4624 (3)	0.36963 (10)	0.0285 (4)	
H1C	0.4186 (17)	0.486 (5)	0.3355 (15)	0.066 (11)*	
H1D	0.4160 (13)	0.558 (4)	0.3874 (11)	0.033 (7)*	
C2	−0.02989 (13)	0.9642 (4)	0.34926 (11)	0.0372 (6)	
H2A	−0.0396	0.9255	0.3097	0.045*	
H2B	−0.0541	1.0774	0.3508	0.045*	
C2'	0.50016 (12)	0.4304 (4)	0.39128 (12)	0.0399 (6)	
H2C	0.5148	0.4459	0.4324	0.048*	
H2D	0.5241	0.5175	0.3743	0.048*	
C3	−0.04994 (12)	0.8175 (4)	0.38393 (11)	0.0385 (6)	
H3A	−0.0485	0.8648	0.4214	0.046*	
H3B	−0.0949	0.7759	0.3653	0.046*	
C3'	0.51319 (13)	0.2377 (4)	0.37573 (13)	0.0402 (6)	
H3C	0.5089	0.2299	0.3352	0.048*	
H3D	0.5580	0.2007	0.3966	0.048*	
N4	−0.00203 (10)	0.6626 (3)	0.38910 (10)	0.0293 (4)	
H4A	−0.0026 (13)	0.589 (4)	0.4168 (12)	0.039 (8)*	
H4B	−0.0154 (14)	0.589 (4)	0.3567 (13)	0.044 (8)*	
N4'	0.46398 (10)	0.1153 (3)	0.39043 (10)	0.0300 (5)	
H4C	0.4779 (15)	0.075 (4)	0.4248 (13)	0.049 (9)*	
H4D	0.4624 (13)	0.019 (4)	0.3731 (11)	0.032 (8)*	
N5	0.12897 (11)	0.5183 (3)	0.41295 (9)	0.0296 (4)	
H5A	0.1141 (13)	0.451 (4)	0.4369 (11)	0.037 (8)*	
H5B	0.1737 (15)	0.547 (4)	0.4276 (11)	0.040 (8)*	
N5'	0.32999 (11)	0.0116 (3)	0.37956 (9)	0.0287 (4)	
H5C	0.2923 (14)	0.045 (4)	0.3883 (11)	0.036 (7)*	
H5D	0.3548 (15)	−0.055 (4)	0.4060 (13)	0.044 (9)*	
C6	0.11746 (13)	0.4154 (3)	0.35841 (11)	0.0329 (5)	
H6A	0.0732	0.3620	0.3474	0.039*	
H6B	0.1498	0.3169	0.3623	0.039*	
C6'	0.31418 (14)	−0.0861 (4)	0.32408 (11)	0.0398 (6)	
H6C	0.2753	−0.1641	0.3195	0.048*	
H6D	0.3514	−0.1631	0.3220	0.048*	
C7	0.12481 (13)	0.5530 (3)	0.31469 (11)	0.0333 (5)	
H7A	0.1707	0.5940	0.3229	0.040*	
H7B	0.1116	0.4987	0.2769	0.040*	
C7'	0.30080 (14)	0.0588 (4)	0.27837 (11)	0.0431 (7)	
H7C	0.2975	0.0036	0.2415	0.052*	
H7D	0.2596	0.1232	0.2763	0.052*	
N8	0.08093 (11)	0.7084 (3)	0.31812 (9)	0.0278 (4)	
H8A	0.0920 (15)	0.810 (5)	0.3010 (13)	0.055 (9)*	
H8B	0.0437 (14)	0.683 (4)	0.3021 (12)	0.037 (8)*	
N8'	0.35805 (12)	0.1872 (3)	0.29494 (9)	0.0331 (5)	
H8C	0.3901 (16)	0.134 (4)	0.2844 (13)	0.051 (9)*	
H8D	0.3526 (15)	0.293 (4)	0.2772 (13)	0.051 (9)*	
N9	0.09587 (11)	0.8161 (3)	0.47994 (8)	0.0305 (5)	
H9A	0.0589 (18)	0.808 (5)	0.4869 (14)	0.068 (11)*	
H9B	0.1073 (15)	0.934 (4)	0.4830 (12)	0.048 (9)*	
N9'	0.39414 (11)	0.2930 (3)	0.45935 (9)	0.0301 (5)	
H9C	0.4328 (15)	0.255 (4)	0.4767 (12)	0.038 (8)*	
H9D	0.3938 (12)	0.410 (4)	0.4623 (10)	0.024 (7)*	
C10	0.14694 (15)	0.7113 (4)	0.52328 (11)	0.0466 (7)	
H10A	0.1869	0.7033	0.5107	0.056*	
H10B	0.1307	0.5866	0.5250	0.056*	
C10'	0.34564 (14)	0.2178 (4)	0.48815 (11)	0.0390 (6)	
H10C	0.3585	0.0928	0.5008	0.047*	
H10D	0.3018	0.2127	0.4609	0.047*	
C11	0.16542 (13)	0.7919 (4)	0.58258 (11)	0.0370 (6)	
C11'	0.34216 (12)	0.3326 (4)	0.53849 (11)	0.0350 (6)	
C12	0.15612 (14)	0.6896 (5)	0.62752 (12)	0.0482 (7)	
H12	0.1346	0.5760	0.6204	0.058*	
C12'	0.35761 (15)	0.2587 (4)	0.59266 (12)	0.0464 (7)	
H12'	0.3706	0.1358	0.5986	0.056*	
C13	0.17863 (16)	0.7549 (6)	0.68334 (13)	0.0595 (10)	
H13	0.1722	0.6855	0.7134	0.071*	
C13'	0.35379 (18)	0.3676 (6)	0.63837 (13)	0.0624 (10)	
H13'	0.3640	0.3167	0.6748	0.075*	
C14	0.20967 (15)	0.9192 (6)	0.69345 (14)	0.0643 (10)	
H14	0.2252	0.9620	0.7307	0.077*	
C14'	0.33530 (17)	0.5479 (6)	0.63053 (15)	0.0631 (10)	
H14'	0.3332	0.6201	0.6615	0.076*	
C15	0.21876 (15)	1.0241 (5)	0.64982 (15)	0.0619 (9)	
H15	0.2400	1.1378	0.6575	0.074*	
C15'	0.31972 (14)	0.6229 (5)	0.57686 (15)	0.0548 (8)	
H15'	0.3071	0.7462	0.5714	0.066*	
C16	0.19618 (15)	0.9606 (4)	0.59400 (13)	0.0487 (7)	
H16	0.2019	1.0326	0.5642	0.058*	
C16'	0.32269 (13)	0.5152 (4)	0.53083 (13)	0.0436 (7)	
H16'	0.3115	0.5662	0.4944	0.052*	

### Atomic displacement parameters (Å^2^)

**Table:**

	*U*^11^	*U*^22^	*U*^33^	*U*^12^	*U*^13^	*U*^23^
Co1	0.01975 (15)	0.02389 (16)	0.02415 (16)	0.00014 (12)	0.00701 (12)	0.00230 (12)
Co1'	0.01926 (15)	0.02633 (16)	0.02270 (16)	−0.00031 (12)	0.00564 (12)	0.00163 (12)
Cl1	0.0292 (3)	0.0464 (4)	0.0532 (4)	−0.0123 (3)	0.0149 (3)	−0.0018 (3)
Cl1'	0.0254 (3)	0.0454 (4)	0.0503 (4)	0.0103 (3)	0.0049 (3)	0.0065 (3)
Cl2	0.0563 (4)	0.0368 (4)	0.0404 (4)	−0.0037 (3)	0.0264 (3)	−0.0006 (3)
Cl2'	0.0416 (4)	0.0367 (3)	0.0360 (3)	0.0009 (3)	0.0028 (3)	0.0037 (3)
Cl3	0.0526 (4)	0.0451 (4)	0.0382 (4)	0.0053 (3)	0.0102 (3)	0.0142 (3)
Cl3'	0.0563 (5)	0.0478 (4)	0.0434 (4)	−0.0077 (3)	0.0172 (3)	0.0094 (3)
O1	0.0703 (18)	0.0750 (19)	0.103 (2)	−0.0202 (15)	0.0519 (17)	−0.0476 (16)
N1	0.0357 (12)	0.0265 (11)	0.0293 (11)	0.0034 (9)	0.0111 (10)	0.0015 (9)
N1'	0.0266 (11)	0.0296 (11)	0.0286 (11)	−0.0022 (8)	0.0065 (9)	0.0016 (9)
C2	0.0335 (14)	0.0399 (15)	0.0340 (14)	0.0148 (11)	0.0028 (11)	−0.0009 (11)
C2'	0.0282 (13)	0.0447 (16)	0.0457 (16)	−0.0104 (12)	0.0085 (12)	−0.0029 (12)
C3	0.0234 (13)	0.0556 (17)	0.0379 (14)	0.0086 (12)	0.0109 (11)	−0.0016 (12)
C3'	0.0272 (13)	0.0449 (16)	0.0527 (17)	−0.0045 (11)	0.0182 (12)	−0.0063 (13)
N4	0.0244 (11)	0.0349 (12)	0.0288 (11)	−0.0010 (9)	0.0082 (9)	0.0050 (9)
N4'	0.0290 (11)	0.0306 (12)	0.0322 (12)	0.0022 (9)	0.0116 (10)	0.0026 (10)
N5	0.0286 (12)	0.0294 (11)	0.0319 (11)	0.0025 (9)	0.0105 (10)	0.0044 (9)
N5'	0.0257 (11)	0.0331 (11)	0.0286 (11)	−0.0027 (9)	0.0096 (9)	0.0020 (9)
C6	0.0326 (13)	0.0277 (12)	0.0387 (14)	0.0045 (10)	0.0105 (11)	−0.0014 (10)
C6'	0.0463 (16)	0.0359 (14)	0.0395 (15)	−0.0155 (12)	0.0159 (12)	−0.0071 (11)
C7	0.0343 (14)	0.0347 (14)	0.0335 (13)	0.0060 (11)	0.0136 (11)	−0.0016 (10)
C7'	0.0444 (16)	0.0530 (18)	0.0294 (14)	−0.0202 (14)	0.0064 (12)	−0.0079 (12)
N8	0.0260 (12)	0.0305 (11)	0.0279 (11)	0.0013 (9)	0.0093 (9)	0.0028 (8)
N8'	0.0373 (13)	0.0347 (12)	0.0274 (11)	−0.0104 (10)	0.0090 (10)	0.0000 (9)
N9	0.0266 (12)	0.0394 (13)	0.0243 (10)	0.0015 (9)	0.0053 (9)	0.0020 (9)
N9'	0.0251 (11)	0.0382 (13)	0.0276 (11)	0.0001 (9)	0.0084 (9)	−0.0004 (9)
C10	0.0485 (18)	0.0532 (18)	0.0299 (14)	0.0133 (14)	−0.0026 (13)	−0.0027 (12)
C10'	0.0430 (16)	0.0448 (16)	0.0352 (14)	−0.0079 (12)	0.0210 (12)	−0.0057 (12)
C11	0.0265 (13)	0.0515 (17)	0.0286 (13)	0.0064 (11)	0.0007 (10)	−0.0020 (11)
C11'	0.0297 (13)	0.0461 (15)	0.0329 (13)	−0.0057 (11)	0.0149 (11)	−0.0035 (11)
C12	0.0353 (16)	0.0614 (19)	0.0449 (17)	0.0061 (14)	0.0062 (13)	0.0066 (14)
C12'	0.0500 (18)	0.0554 (18)	0.0387 (15)	−0.0091 (14)	0.0206 (14)	0.0021 (13)
C13	0.0387 (17)	0.106 (3)	0.0333 (16)	0.0176 (19)	0.0089 (13)	0.0153 (17)
C13'	0.069 (2)	0.090 (3)	0.0370 (17)	−0.026 (2)	0.0282 (16)	−0.0092 (17)
C14	0.0352 (17)	0.117 (3)	0.0351 (17)	0.0052 (19)	0.0018 (14)	−0.0164 (19)
C14'	0.055 (2)	0.090 (3)	0.058 (2)	−0.0272 (19)	0.0379 (18)	−0.0359 (19)
C15	0.0395 (18)	0.080 (3)	0.062 (2)	−0.0099 (16)	0.0085 (16)	−0.0290 (19)
C15'	0.0350 (16)	0.059 (2)	0.077 (2)	−0.0031 (14)	0.0271 (16)	−0.0213 (17)
C16	0.0410 (17)	0.061 (2)	0.0433 (17)	0.0004 (14)	0.0109 (13)	−0.0036 (14)
C16'	0.0290 (14)	0.0562 (18)	0.0456 (16)	0.0028 (12)	0.0105 (12)	−0.0015 (13)

### Geometric parameters (Å, °)

**Table:**

Co1—N1	1.933 (2)	C6'—H6C	0.9700
Co1—N4	1.950 (2)	C6'—H6D	0.9700
Co1—N8	1.954 (2)	C7—N8	1.471 (3)
Co1—N5	1.959 (2)	C7—H7A	0.9700
Co1—N9	2.001 (2)	C7—H7B	0.9700
Co1—Cl1	2.2592 (7)	C7'—N8'	1.480 (3)
Co1'—N1'	1.942 (2)	C7'—H7C	0.9700
Co1'—N4'	1.948 (2)	C7'—H7D	0.9700
Co1'—N8'	1.959 (2)	N8—H8A	0.91 (3)
Co1'—N5'	1.962 (2)	N8—H8B	0.79 (3)
Co1'—N9'	1.989 (2)	N8'—H8C	0.87 (3)
Co1'—Cl1'	2.2415 (7)	N8'—H8D	0.87 (3)
O1—H1W	0.84 (3)	N9—C10	1.483 (3)
O1—H2W	0.842 (17)	N9—H9A	0.84 (4)
N1—C2	1.478 (3)	N9—H9B	0.89 (3)
N1—H1A	0.81 (3)	N9'—C10'	1.492 (3)
N1—H1B	0.87 (3)	N9'—H9C	0.85 (3)
N1'—C2'	1.468 (3)	N9'—H9D	0.85 (3)
N1'—H1C	0.82 (3)	C10—C11	1.507 (4)
N1'—H1D	0.89 (3)	C10—H10A	0.9700
C2—C3	1.491 (4)	C10—H10B	0.9700
C2—H2A	0.9700	C10'—C11'	1.503 (3)
C2—H2B	0.9700	C10'—H10C	0.9700
C2'—C3'	1.492 (4)	C10'—H10D	0.9700
C2'—H2C	0.9700	C11—C16	1.373 (4)
C2'—H2D	0.9700	C11—C12	1.384 (4)
C3—N4	1.487 (3)	C11'—C12'	1.379 (4)
C3—H3A	0.9700	C11'—C16'	1.381 (4)
C3—H3B	0.9700	C12—C13	1.394 (4)
C3'—N4'	1.479 (3)	C12—H12	0.9300
C3'—H3C	0.9700	C12'—C13'	1.388 (4)
C3'—H3D	0.9700	C12'—H12'	0.9300
N4—H4A	0.87 (3)	C13—C14	1.345 (5)
N4—H4B	0.93 (3)	C13—H13	0.9300
N4'—H4C	0.86 (3)	C13'—C14'	1.361 (5)
N4'—H4D	0.81 (3)	C13'—H13'	0.9300
N5—C6	1.486 (3)	C14—C15	1.366 (5)
N5—H5A	0.88 (3)	C14—H14	0.9300
N5—H5B	0.93 (3)	C14'—C15'	1.371 (5)
N5'—C6'	1.481 (3)	C14'—H14'	0.9300
N5'—H5C	0.91 (3)	C15—C16	1.389 (4)
N5'—H5D	0.85 (3)	C15—H15	0.9300
C6—C7	1.500 (3)	C15'—C16'	1.385 (4)
C6—H6A	0.9700	C15'—H15'	0.9300
C6—H6B	0.9700	C16—H16	0.9300
C6'—C7'	1.500 (4)	C16'—H16'	0.9300
			
N1—Co1—N4	85.69 (9)	N5—C6—H6B	110.4
N1—Co1—N8	88.81 (9)	C7—C6—H6B	110.4
N4—Co1—N8	91.65 (10)	H6A—C6—H6B	108.6
N1—Co1—N5	173.44 (9)	N5'—C6'—C7'	107.0 (2)
N4—Co1—N5	93.07 (9)	N5'—C6'—H6C	110.3
N8—Co1—N5	84.78 (9)	C7'—C6'—H6C	110.3
N1—Co1—N9	91.95 (10)	N5'—C6'—H6D	110.3
N4—Co1—N9	89.85 (10)	C7'—C6'—H6D	110.3
N8—Co1—N9	178.36 (9)	H6C—C6'—H6D	108.6
N5—Co1—N9	94.48 (9)	N8—C7—C6	106.04 (19)
N1—Co1—Cl1	90.06 (7)	N8—C7—H7A	110.5
N4—Co1—Cl1	175.24 (7)	C6—C7—H7A	110.5
N8—Co1—Cl1	90.41 (7)	N8—C7—H7B	110.5
N5—Co1—Cl1	91.40 (7)	C6—C7—H7B	110.5
N9—Co1—Cl1	88.14 (7)	H7A—C7—H7B	108.7
N1'—Co1'—N4'	85.16 (9)	N8'—C7'—C6'	105.7 (2)
N1'—Co1'—N8'	92.50 (9)	N8'—C7'—H7C	110.6
N4'—Co1'—N8'	90.53 (11)	C6'—C7'—H7C	110.6
N1'—Co1'—N5'	176.43 (9)	N8'—C7'—H7D	110.6
N4'—Co1'—N5'	93.27 (9)	C6'—C7'—H7D	110.6
N8'—Co1'—N5'	84.30 (9)	H7C—C7'—H7D	108.7
N1'—Co1'—N9'	89.81 (10)	C7—N8—Co1	110.07 (15)
N4'—Co1'—N9'	92.02 (10)	C7—N8—H8A	110 (2)
N8'—Co1'—N9'	176.70 (9)	Co1—N8—H8A	110 (2)
N5'—Co1'—N9'	93.45 (9)	C7—N8—H8B	110 (2)
N1'—Co1'—Cl1'	89.14 (7)	Co1—N8—H8B	108 (2)
N4'—Co1'—Cl1'	174.30 (7)	H8A—N8—H8B	108 (3)
N8'—Co1'—Cl1'	89.78 (8)	C7'—N8'—Co1'	109.39 (16)
N5'—Co1'—Cl1'	92.42 (7)	C7'—N8'—H8C	106 (2)
N9'—Co1'—Cl1'	87.90 (7)	Co1'—N8'—H8C	115 (2)
H1W—O1—H2W	106 (4)	C7'—N8'—H8D	116 (2)
C2—N1—Co1	110.22 (16)	Co1'—N8'—H8D	107 (2)
C2—N1—H1A	111 (2)	H8C—N8'—H8D	104 (3)
Co1—N1—H1A	111 (2)	C10—N9—Co1	117.35 (17)
C2—N1—H1B	111.4 (17)	C10—N9—H9A	110 (2)
Co1—N1—H1B	111.2 (17)	Co1—N9—H9A	111 (2)
H1A—N1—H1B	102 (3)	C10—N9—H9B	108.2 (19)
C2'—N1'—Co1'	110.90 (16)	Co1—N9—H9B	102.8 (19)
C2'—N1'—H1C	109 (2)	H9A—N9—H9B	107 (3)
Co1'—N1'—H1C	106 (2)	C10'—N9'—Co1'	118.23 (17)
C2'—N1'—H1D	110.6 (17)	C10'—N9'—H9C	109.1 (19)
Co1'—N1'—H1D	111.5 (17)	Co1'—N9'—H9C	107.7 (19)
H1C—N1'—H1D	108 (3)	C10'—N9'—H9D	107.5 (16)
N1—C2—C3	107.2 (2)	Co1'—N9'—H9D	105.7 (16)
N1—C2—H2A	110.3	H9C—N9'—H9D	108 (2)
C3—C2—H2A	110.3	N9—C10—C11	115.2 (2)
N1—C2—H2B	110.3	N9—C10—H10A	108.5
C3—C2—H2B	110.3	C11—C10—H10A	108.5
H2A—C2—H2B	108.5	N9—C10—H10B	108.5
N1'—C2'—C3'	107.4 (2)	C11—C10—H10B	108.5
N1'—C2'—H2C	110.2	H10A—C10—H10B	107.5
C3'—C2'—H2C	110.2	N9'—C10'—C11'	112.4 (2)
N1'—C2'—H2D	110.2	N9'—C10'—H10C	109.1
C3'—C2'—H2D	110.2	C11'—C10'—H10C	109.1
H2C—C2'—H2D	108.5	N9'—C10'—H10D	109.1
N4—C3—C2	107.13 (19)	C11'—C10'—H10D	109.1
N4—C3—H3A	110.3	H10C—C10'—H10D	107.9
C2—C3—H3A	110.3	C16—C11—C12	118.6 (3)
N4—C3—H3B	110.3	C16—C11—C10	121.2 (3)
C2—C3—H3B	110.3	C12—C11—C10	120.0 (3)
H3A—C3—H3B	108.5	C12'—C11'—C16'	118.8 (3)
N4'—C3'—C2'	108.0 (2)	C12'—C11'—C10'	121.2 (3)
N4'—C3'—H3C	110.1	C16'—C11'—C10'	120.0 (2)
C2'—C3'—H3C	110.1	C11—C12—C13	120.7 (3)
N4'—C3'—H3D	110.1	C11—C12—H12	119.7
C2'—C3'—H3D	110.1	C13—C12—H12	119.7
H3C—C3'—H3D	108.4	C11'—C12'—C13'	120.0 (3)
C3—N4—Co1	109.37 (17)	C11'—C12'—H12'	120.0
C3—N4—H4A	112.0 (18)	C13'—C12'—H12'	120.0
Co1—N4—H4A	111.5 (18)	C14—C13—C12	119.5 (3)
C3—N4—H4B	109.0 (18)	C14—C13—H13	120.3
Co1—N4—H4B	110.7 (17)	C12—C13—H13	120.3
H4A—N4—H4B	104 (3)	C14'—C13'—C12'	120.8 (3)
C3'—N4'—Co1'	110.18 (16)	C14'—C13'—H13'	119.6
C3'—N4'—H4C	112 (2)	C12'—C13'—H13'	119.6
Co1'—N4'—H4C	112 (2)	C13—C14—C15	121.1 (3)
C3'—N4'—H4D	108.3 (19)	C13—C14—H14	119.5
Co1'—N4'—H4D	114.4 (19)	C15—C14—H14	119.5
H4C—N4'—H4D	100 (3)	C13'—C14'—C15'	119.7 (3)
C6—N5—Co1	109.37 (15)	C13'—C14'—H14'	120.1
C6—N5—H5A	108.6 (17)	C15'—C14'—H14'	120.1
Co1—N5—H5A	113.4 (17)	C14—C15—C16	119.9 (3)
C6—N5—H5B	110.4 (17)	C14—C15—H15	120.1
Co1—N5—H5B	102.9 (17)	C16—C15—H15	120.1
H5A—N5—H5B	112 (2)	C14'—C15'—C16'	120.0 (3)
C6'—N5'—Co1'	110.85 (15)	C14'—C15'—H15'	120.0
C6'—N5'—H5C	111.1 (17)	C16'—C15'—H15'	120.0
Co1'—N5'—H5C	106.0 (17)	C11—C16—C15	120.3 (3)
C6'—N5'—H5D	110.8 (19)	C11—C16—H16	119.9
Co1'—N5'—H5D	107.1 (19)	C15—C16—H16	119.9
H5C—N5'—H5D	111 (3)	C11'—C16'—C15'	120.6 (3)
N5—C6—C7	106.4 (2)	C11'—C16'—H16'	119.7
N5—C6—H6A	110.4	C15'—C16'—H16'	119.7
C7—C6—H6A	110.4		
			
N4—Co1—N1—C2	−13.68 (17)	N4—Co1—N8—C7	−108.29 (17)
N8—Co1—N1—C2	78.07 (18)	N5—Co1—N8—C7	−15.35 (17)
N5—Co1—N1—C2	65.6 (9)	N9—Co1—N8—C7	48 (3)
N9—Co1—N1—C2	−103.38 (18)	Cl1—Co1—N8—C7	76.01 (16)
Cl1—Co1—N1—C2	168.47 (17)	C6'—C7'—N8'—Co1'	−44.3 (3)
N4'—Co1'—N1'—C2'	−13.81 (18)	N1'—Co1'—N8'—C7'	−161.0 (2)
N8'—Co1'—N1'—C2'	−104.13 (19)	N4'—Co1'—N8'—C7'	113.9 (2)
N5'—Co1'—N1'—C2'	−77.8 (16)	N5'—Co1'—N8'—C7'	20.6 (2)
N9'—Co1'—N1'—C2'	78.23 (19)	N9'—Co1'—N8'—C7'	−26.6 (19)
Cl1'—Co1'—N1'—C2'	166.13 (17)	Cl1'—Co1'—N8'—C7'	−71.82 (19)
Co1—N1—C2—C3	38.0 (2)	N1—Co1—N9—C10	−160.1 (2)
Co1'—N1'—C2'—C3'	36.6 (3)	N4—Co1—N9—C10	114.2 (2)
N1—C2—C3—N4	−48.9 (3)	N8—Co1—N9—C10	−42 (3)
N1'—C2'—C3'—N4'	−46.5 (3)	N5—Co1—N9—C10	21.1 (2)
C2—C3—N4—Co1	37.9 (2)	Cl1—Co1—N9—C10	−70.1 (2)
N1—Co1—N4—C3	−13.78 (17)	N1'—Co1'—N9'—C10'	154.6 (2)
N8—Co1—N4—C3	−102.47 (17)	N4'—Co1'—N9'—C10'	−120.3 (2)
N5—Co1—N4—C3	172.67 (17)	N8'—Co1'—N9'—C10'	20.1 (19)
N9—Co1—N4—C3	78.19 (17)	N5'—Co1'—N9'—C10'	−26.9 (2)
Cl1—Co1—N4—C3	13.1 (9)	Cl1'—Co1'—N9'—C10'	65.4 (2)
C2'—C3'—N4'—Co1'	35.6 (3)	Co1—N9—C10—C11	163.4 (2)
N1'—Co1'—N4'—C3'	−12.49 (19)	Co1'—N9'—C10'—C11'	−152.63 (19)
N8'—Co1'—N4'—C3'	79.97 (19)	N9—C10—C11—C16	−64.9 (4)
N5'—Co1'—N4'—C3'	164.29 (18)	N9—C10—C11—C12	120.1 (3)
N9'—Co1'—N4'—C3'	−102.13 (19)	N9'—C10'—C11'—C12'	−119.6 (3)
Cl1'—Co1'—N4'—C3'	−13.1 (9)	N9'—C10'—C11'—C16'	60.9 (3)
N1—Co1—N5—C6	−1.6 (9)	C16—C11—C12—C13	−1.2 (4)
N4—Co1—N5—C6	77.27 (17)	C10—C11—C12—C13	174.0 (3)
N8—Co1—N5—C6	−14.11 (17)	C16'—C11'—C12'—C13'	−0.3 (4)
N9—Co1—N5—C6	167.36 (17)	C10'—C11'—C12'—C13'	−179.8 (3)
Cl1—Co1—N5—C6	−104.39 (16)	C11—C12—C13—C14	0.0 (5)
N1'—Co1'—N5'—C6'	−18.2 (16)	C11'—C12'—C13'—C14'	−0.4 (5)
N4'—Co1'—N5'—C6'	−82.01 (19)	C12—C13—C14—C15	0.8 (5)
N8'—Co1'—N5'—C6'	8.20 (19)	C12'—C13'—C14'—C15'	0.5 (5)
N9'—Co1'—N5'—C6'	−174.23 (19)	C13—C14—C15—C16	−0.5 (5)
Cl1'—Co1'—N5'—C6'	97.73 (18)	C13'—C14'—C15'—C16'	0.1 (5)
Co1—N5—C6—C7	39.8 (2)	C12—C11—C16—C15	1.5 (4)
Co1'—N5'—C6'—C7'	−34.7 (3)	C10—C11—C16—C15	−173.6 (3)
N5—C6—C7—N8	−52.0 (3)	C14—C15—C16—C11	−0.7 (5)
N5'—C6'—C7'—N8'	50.7 (3)	C12'—C11'—C16'—C15'	1.0 (4)
C6—C7—N8—Co1	40.9 (2)	C10'—C11'—C16'—C15'	−179.5 (2)
N1—Co1—N8—C7	166.07 (18)	C14'—C15'—C16'—C11'	−0.9 (4)

### Hydrogen-bond geometry (Å, °)

**Table:**

*D*—H···*A*	*D*—H	H···*A*	*D*···*A*	*D*—H···*A*
N1—H1A···Cl3	0.81 (3)	2.67 (3)	3.406 (2)	151 (3)
N1—H1B···Cl2	0.87 (3)	2.36 (3)	3.211 (2)	163 (2)
N8—H8A···Cl3	0.91 (3)	2.33 (3)	3.179 (2)	156 (3)
N4'—H4D···O1	0.81 (3)	2.25 (3)	2.915 (3)	139 (2)
N8'—H8C···O1	0.87 (3)	2.15 (3)	2.964 (4)	155 (3)
N1'—H1C···Cl3'	0.82 (3)	2.43 (4)	3.246 (2)	174 (3)
N4—H4A···Cl2^i^	0.87 (3)	2.65 (3)	3.423 (2)	149 (2)
N5'—H5D···Cl2'	0.85 (3)	2.39 (3)	3.233 (2)	168 (3)
N8'—H8D···Cl3'	0.87 (3)	2.48 (3)	3.252 (2)	148 (3)
N4—H4B···Cl3^ii^	0.93 (3)	2.45 (3)	3.265 (2)	147 (2)
N8—H8B···Cl3^ii^	0.79 (3)	2.49 (3)	3.280 (2)	176 (3)
N9—H9A···Cl2^iii^	0.84 (4)	2.57 (4)	3.391 (2)	166 (3)
N5'—H5C···Cl1^i^	0.91 (3)	2.63 (3)	3.380 (2)	141 (2)
N1'—H1D···Cl2'^iv^	0.89 (3)	2.49 (3)	3.288 (2)	149 (2)
N9'—H9D···Cl2'^iv^	0.85 (3)	2.81 (3)	3.615 (2)	158 (2)
N5—H5A···Cl2^i^	0.88 (3)	2.38 (3)	3.220 (2)	159 (2)
O1—H2W···Cl3'^i^	0.84 (2)	2.27 (2)	3.092 (3)	165 (4)

Symmetry codes:  (i) *x*, *y*−1, *z*; (ii) −*x*, *y*−1/2, −*z*+1/2; (iii) −*x*, −*y*+2, −*z*+1; (iv) *x*, *y*+1, *z*.
